# Evidence of Autochthonous Human *Strongyloides stercoralis* in South Carolina

**DOI:** 10.4269/ajtmh.25-0209

**Published:** 2025-08-19

**Authors:** Matthew S. Haldeman, Henry Heidt, Salomé-Joëlle Gass, Melissa S. Nolan

**Affiliations:** ^1^ Department of Family and Preventive Medicine, School of Medicine–Columbia, University of South Carolina, Columbia, South Carolina;; ^2^ Department of Epidemiology and Biostatistics, Arnold School of Public Health, University of South Carolina, Columbia, South Carolina;; ^3^ Department of Health Service, Policy, and Management, Arnold School of Public Health, University of South Columbia, South Carolina

## Abstract

*Strongyloides stercoralis*, a soil-transmitted helminth, was historically known to persist throughout the southeastern United States, but data regarding current prevalence are lacking. This project aimed to evaluate potential seroprevalence and epidemiologic risk factors for *Strongyloides* infections among South Carolina (SC) residents. First, a cross-sectional serosurvey was conducted on banked serum samples, and seroprevalence status was compared with associated health questionnaires. Seropositive participants were contacted for follow-up confirmation and management. Second, a retrospective chart abstraction was performed within the state’s largest health care system, comparing medical records among patients testing positive for *Strongyloides* with two matched controls over a 5-year period. From the initial cross-sectional serosurvey, 5.0% (*n* = 78 of 1,572) of sera tested positive for *Strongyloides* antibodies. Significant differences in race/ethnicity and education level were noted between positive and negative residents. Geospatial analysis revealed statistical hot spots in northwestern and central SC. Follow-up participation of seropositives was low (*n* = 14 of 78); however, five of these participants (36%) were positive on confirmation testing. Of these, three had evidence of autochthonous transmission. Conversely, the retrospective chart abstraction revealed 26 patients with confirmed laboratory diagnosis of *Strongyloides* infection, of which 6 had evidence of autochthonous transmission. We found a small but nonnegligible burden of autochthonous *Strongyloides* infection among SC residents. Further study is needed to better characterize the clinical burden and epidemiologic risk factors for locally acquired *S. stercoralis*. This information may provide contemporary data to inform future targeted public health interventions for at-risk populations in the southeastern United States.

## INTRODUCTION

Soil-transmitted helminths (STHs), a group of human parasitic nematodes, affect up to one third of the global population, persisting primarily among impoverished populations in low-resource settings.^1^^,^^2^ Given their ability to cause chronic malnutrition and anemia, especially in children, STH infection has been associated with reduced cognitive functioning, poor educational achievement, and lower work output in infected populations, potentiating the cycle of poverty.^2^^–^^4^ In the United States, STHs historically have caused the largest disease burden in the southern and Appalachia Mountain range regions given these regions’ suitable climate and high burden of rural poverty among other factors.^2^^–^^4^ Historically, extensive public health campaigns, such as the Rockefeller Sanitary Commission, were undertaken to control STHs nationally, but widespread surveillance ceased after the 1980s, despite little data demonstrating that elimination had been achieved.^4^ In the past decade, limited studies suggest potential persistence of STH parasitic infection in these regions,^3^^–^^5^ but in the absence of robust surveillance data and because of the subclinical nature of STH infections complicating diagnosis, little is known regarding the current STH disease burden in the southern United States.^2^^,^^4^

*Strongyloides stercoralis* is a clinically important STH as this subclinical or mild infection can propagate inside a host through autoinfection pathogenic mechanisms, and if activated by corticosteroids or immunosuppressing conditions, hyperinfection and disseminated disease can be fatal.^6^ Limited environmental (soil) and pediatric surveillance studies in impoverished communities have indicated focal *S. stercoralis* human transmission in Texas, Kentucky, Mississippi, and Alabama.^7^^–^^11^ Additionally, isolated infections in Pennsylvania and Arizona have been reported: a case report of a previously healthy child with environmental exposure in Pennsylvania and an isolated outbreak among developmentally disabled residents with presumed poor hygienic practices in a long-term care facility in Arizona.^12^^,^^13^ Despite these case reports, negative surveillance studies among similar at-risk southeastern U.S. populations exist, highlighting the complex and elusive nature of *S. stercoralis* infection nationally.^14^^–^^16^

South Carolina (SC) is a southeastern state with historically high STH rates.^1^^,^^2^ The last formal SC-based human surveillance effort was conducted in 1972, when investigators identified moderate levels of STH infection (1.1–21.5%) among coastal school-aged children, but there was an absence of *S. stercoralis* infection.^4^ This effort followed a 1969 statewide study in which 2–13% of stool-based examinations had STH eggs microscopically identified, with focal geospatial clusters noted; 73% of children examined in Bluffton, SC had positive stool results.^3^ Approximately 47 years after this sampling effort, soil sample studies found that 5% of samples from two different sample locations in southwestern SC were positive for *S. stercoralis* parasitic DNA (overall STH parasitic burden of 28% across five SC sites).^5^ During this same decade, a confirmed clinical *Ascaris* case was identified in a New York resident who reported frequent visits to SC, where clinicians suspected his infection originated,^17^ highlighting potential contemporary human transmission in the state.

Despite robust historical STH surveillance, little is known regarding the current STH burden, including *S. stercoralis*, in the rural southern United States.^2^^,^^4^ Given SC’s collective built environment and climatic factors supportive of *S. stercoralis* transmission and historical endemicity, potential for contemporary focal transmission cycles exists. Therefore, the purpose of this study was to evaluate potential autochthonous transmission, epidemiologic profiles, and clinical impact using a two-pronged study design approach to inform future efforts for prospective public health intervention.

## MATERIALS AND METHODS

**Figure 1.**
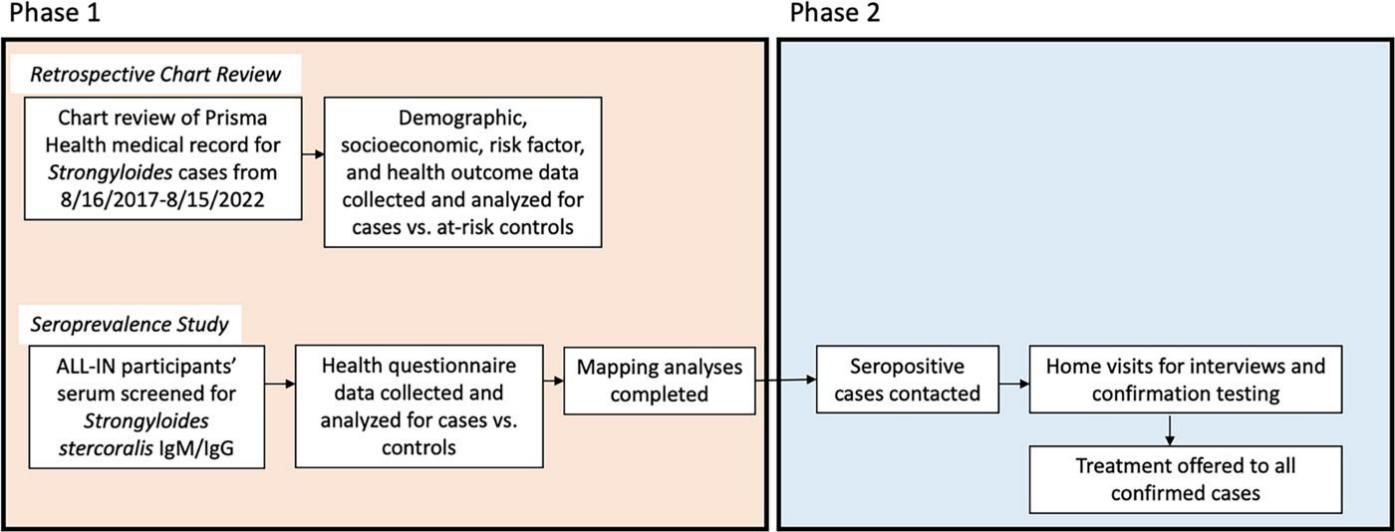
Pictorial overview of study methodology. ALL-IN: A Learning Laboratory Innovative, National, Self-Collection Study.

This study used a combined strategy involving both active and passive surveillance methods to estimate current *S. stercoralis* prevalence and describe associated clinical–epidemiologic profiles. Both studies were reviewed and approved by the institutional review boards at the University of South Carolina (Protocol nos. 131714 and 117562) and Prisma Health (Protocol no. 1,937,082). University of South Carolina is the state’s flagship university principally located in Columbia, SC, with an additional seven satellite campuses across the state (total of >56,000 students). Prisma Health is the primary health care system in SC, covering 21 counties and ∼30,000 clinical care staff providing medical services to more than half of the state’s residents. To address the study’s aims, we performed a two-pronged study design (Figure 1). First, a retrospective chart review of *S. stercoralis* cases and controls from Prisma Health was performed to assess the clinical impact of this infection on the local health system. Second, a cross-sectional serology study of biobanked samples collected from a prior coronavirus disease 2019 (COVID-19) study in years 2022–2023 was executed. Any positive persons from this initial cross-section seroprevalence study were then contacted and invited to take part in a second sampling and testing event in 2024.

### Retrospective chart review.

To complete the retrospective chart review, investigators queried the Prisma Health Epic (Verona, WI) electronic medical record for individuals of any age whose records from the 5-year period (August 16, 2017 to August 15, 2022) were associated with the following International Classification of Diseases 10th revision diagnoses: B78.0 (intestinal strongyloidiasis), B78.1. (cutaneous strongyloidiasis), B78.7 (disseminated strongyloidiasis), and/or B78.9 (strongyloidiasis, unspecified). Records from two groups of individuals were eligible for inclusion. Any individual from this time period who was assigned one of these diagnoses was included as a positive case. Individuals from the same time period who were tested for *S. stercoralis* but were negative were included as at-risk controls. Patient charts were not excluded based on race/ethnicity, gender, or other demographic variables.

A standardized chart abstraction form was used to review each patient record and its associated variables, including age, gender, race, ethnicity, zip code, occupation, significant time spent outdoors (if noted), health insurance status (private versus public versus self-pay), alcohol use, provider-determined local exposure, travel exposure, foreign-born status, specific comorbidities (evidence of malnutrition, HIV status, asthma, or other immunocompromising condition), laboratory orders (*Strongyloides* IgM and/or IgG levels, stool ova, and parasite analysis), and patient outcome if hospitalized (admission to intensive care unit and inpatient mortality). The same data were collected using the standardized chart abstraction form for positive cases and at-risk controls. Statistical analysis was performed using χ^2^ tests utilizing Stata v. 18 (College Station, TX).

### Biobanked seroprevalence study.

The seroprevalence analysis was performed using a subset of frozen serum samples obtained previously from the A Learning Laboratory: Innovative, National, Self-Collection Study (ALL-IN) in South Carolina (https://allin-sc.org/), which evaluated perceptions and obstacles to COVID-19 self-testing among residents across the state. Over the period of April 2022 to September 2023, ALL-IN study participants completed both a health questionnaire and a blood sample donation. The original health questionnaire asked participants about their age, gender, race/ethnicity, income level, highest education achieved, occupation, rural versus urban residence, time spent outdoors, and history of asthma. The original blood sample collection was performed by ALL-IN study phlebotomists. Samples were collected in serum separator tubes (SSTs), centrifuged the same day, and stored in a –80°C freezer, where they remained until needed for analysis. Aliquots of these samples were tested for the presence of *S. stercoralis* antibodies using Abcam (Cambridge, United Kingdom) *Strongyloides* IgG/IgM ELISA test kits. Laboratory retesting of equivocals was performed in accordance with manufacturer instructions. Retested equivocal samples that were negative or equivocal a second time were considered negative. Statistical analysis of positive cases and correlated risk factors (from the original study’s questionnaire) was performed using χ^2^ tests utilizing Stata v. 18.

For geospatial analysis, the locations of *S. stercoralis* antibody-positive and -negative residents were mapped using the zip codes of participants’ home addresses in RStudio (R Foundation for Statistical Computing, Vienna, Austria). If a home address zip code was outside SC or identified as invalid by the R package zipcodeR, the zip code of the serum sample collection site was used as a proxy indicator. An optimized hot spot analysis was conducted in ArcGIS Pro (ESRI, Redlands, CA) using Getis-Ord Gi* statistics to identify spatial clusters of positive and negative patients. This analysis detects statistically significant hot spots and cold spots by aggregating incident data and determining the optimal distance band (14,255 meters in this case). It calculates *z*-scores and *P*-values to test spatial randomness and applies false discovery rate correction to account for multiple testing or spatial dependency. Features are weighted based on similarity with neighbors, with 107 statistically significant features identified in this study.

### Follow-up of participants with a seropositive biobanked sample.

Any ALL-IN participants who had positive *S. stercoralis* serology testing were contacted by mail, telephone call, and e-mail (if e-mail address was known) to explain their test results and arrange in-person follow-up meetings. Study staff sent an initial personalized letter and e-mail that introduced the follow-up study, provided basic *S. stercoralis-*related information, discussed their positive screening test result, and provided information for follow-up. If no response was received for 2 weeks postmailing, study staff attempted to call participants with this same information. All seropositive ALL-IN participants were called a minimum of three times, with messages left if possible. For those who could be contacted and agreed to meet, study staff traveled to their homes or location of the participant’s choosing and completed an in-depth survey as well as a contemporary blood, stool, and urine sample collection for *S. stercoralis* confirmation testing. The survey asked questions regarding details of participants’ residence; health-related questions, such as comorbidities, substance use, and unexplained symptoms; and exposures, including time spent outdoors, barefoot walking practices, home sanitation, and international travel history. After survey completion, study phlebotomists obtained blood in an SST vial and urine sample in a Zymo Research DNA/RNA Shield (Ιrvine, CA) preservative. A Zymo DNA/RNA Shield stool preservation container was left with the participant, who mailed fecal material collected in a convenience sampling manner back to the study laboratory in a prepaid overnight shipping package. Serum samples were evaluated for *Strongyloides* serology using Abcam *Strongyloides* IgG/IgM ELISA test kits, whereas urine and stool samples were analyzed for *Strongyloides* polymerase chain reaction (PCR) using Zymo Research DNA Extraction Kits and custom Integrated DNA Technologies primers/probes. Reverse transcription polymerase chain reaction (RT-PCR) testing defined positive samples as cycle threshold values of <40. Given the team’s prior experience with discordant test results (from the biobanked seroprevalence study) and the ample follow-up visit serum sample volume, follow-up participant’s sera were run twice in triplicate for a total of six ELISA results, and urine and stool RT-PCR was run in triplicate.

## RESULTS

### Retrospective chart review.

For the chart abstraction, 153 patient charts were reviewed, comprising 26 cases and 127 controls based on inclusion criteria. All patients reviewed were tested with *S. stercoralis* serologies; none were tested with stool ova testing. Cases were significantly more likely than controls to be Black or White (53.9% combined versus 47.2%; *P* = 0.034) and to have local exposure (23.1% versus 0%; *P* <0.001) (Table 1). Patients tested who were not Black or White were principally Latinx or Middle Eastern immigrants receiving standard in-take clinical management workups in the refugee and migrant health clinic. No additional demographic or parasitic exposures were statistically different between cases and controls. Statistical significance was close for unemployment status (34.6% [cases] versus 49.6% [controls], *P* <0.082) and immunocompromising conditions (11.5% [cases] versus 26.0% [controls], *P* <0.053).

**Table 1:** Risk factors and exposures among *Strongyloides stercoralis*-positive and -negative persons in either the retrospective chart abstraction or biobanked seroprevalence studies

Factors		Prisma Health Chart Abstraction Study				ALL-IN SC Biobanked Seroprevalence Study			
		Total Patient Charts Analyzed	*Strongyloides stercoralis*- Positive Patients	*Strongyloides stercoralis*- Negative Patients	*P*-Value	Total Unique Participant Sera Samples	*Strongyloides stercoralis*- Positive Participants	*Strongyloides stercoralis*- Negative Participants	*P*-Value
		*N* = 153	*N* = 26 (17.0%)	*N* = 127 (83.0%)		*N* = 1,572	*n* = 78 (5.0%)	*n* = 1,494 (95.0%)	
Age, years	<18	22 (14.38%)	4 (15.38%)	18 (14.17%)	0.874	70 (4.45%)	3 (3.85%)	67 (4.48%)	0.210
	18–24	53 (34.64%)	8 (30.77%)	45 (35.43%)		586 (37.28%)	24 (30.77%)	562 (37.62%)	
	25–34	49 (32.03%)	10 (38.46%)	39 (30.71%)		581 (36.96%)	26 (33.33%)	555 (37.15%)	
	65+	29 (18.95%)	4 (15.38%)	25 (19.69%)		334 (21.25%)	25 (32.05%)	309 (20.68%)	
	Unknown	–				1 (0.06%)	0 (0.00%)	1 (0.07%)	
Gender	Female	75 (49.02%)	10 (38.46%)	65 (51.18%)	0.285	985 (62.66%)	48 (61.54%)	937 (62.72%)	0.759
	Male	78 (50.98%)	16 (61.54%)	62 (48.82%)		578 (36.77%)	30 (38.46%)	548 (36.68%)	
	Other	–				9 (0.57%)	0 (0.00%)	9 (0.60%)	
Race/ethnicity	White	63 (41.18%)	14 (53.85%)	49 (38.58%)	0.034	248 (15.78%)	18 (23.08%)	230 (15.39%)	<0.001
	Black	15 (9.80%)	4 (15.38%)	11 (8.66%)		382 (24.30%)	32 (41.03%)	350 (23.43%)	
	Hispanic	18 (11.76%)	5 (19.23%)	13 (10.24%)		22 (1.40%)	2 (2.56%)	20 (1.34%)	
	Other	28 (18.30%)	1 (3.85%)	27 (21.26%)		871 (55.41%)	23 (29.49%)	848 (56.76%)	
	Not reported	29 (18.95%)	2 (7.69%)	27 (21.26%)		49 (3.12%)	3 (3.85%)	46 (3.08%)	
Area of residence	Urban	144 (94.12%)	23 (88.46%)	121 (95.28%)	0.181	1,127 (71.69%)	57 (73.08%)	1,070 (71.62%)	0.854
	Rural	9 (5.88%)	3 (11.54%)	6 (4.72%)		440 (27.99%)	21 (26.92%)	419 (28.05%)	
	Unknown	–				5 (0.32%)	0 (0.00%)	5 (0.33%)	
Annual household income, $	<15,000	–				318 (20.23%)	14 (17.95%)	304 (20.35%)	0.014
	15,000–50,000	–				420 (26.72%)	11 (14.10%)	409 (27.38%)	
	50,000–100,000	–				224 (14.25%)	17 (21.79%)	207 (13.86%)	
	>100,000	–				139 (8.84%)	5 (6.41%)	134 (8.97%)	
	Unknown	–				471 (29.96%)	31 (39.74%)	440 (29.45%)	
Education	Some high school	–				191 (12.15%)	12 (15.38%)	179 (11.98%)	0.037
	High school degree	–				442 (28.12%)	23 (29.49%)	419 (28.05%)	
	Some college	–				303 (19.27%)	8 (10.26%)	295 (19.75%)	
	Associate’s degree	–				105 (6.68%)	1 (1.28%)	104 (6.96%)	
	Bachelor’s degree	–				170 (10.81%)	9 (11.54%)	161 (10.78%)	
	Graduate degree or above	–				175 (11.13%)	9 (11.54%)	166 (11.11%)	
	Unknown	–				186 (11.83%)	16 (20.51%)	170 (11.38%)	
Occupation	Employed	34 (22.22%)	6 (23.08%)	28 (22.05%)	0.082	583 (37.09%)	21 (26.92%)	562 (37.62%)	0.210
	Student	1 (0.65%)	0 (0.00%)	1 (0.79%)		233 (14.82%)	10 (12.82%)	223 (14.93%)	
	Retired	29 (18.95%)	5 (19.23%)	24 (18.90%)		295 (18.77%)	18 (23.08%)	277 (18.54%)	
	Unemployed	72 (47.06%)	9 (34.62%)	63 (49.61%)		203 (12.91%)	9 (11.54%)	194 (12.99%)	
	Other	8 (5.23%)	1 (3.85%)	7 (5.51%)		69 (4.39%)	5 (6.41%)	64 (4.28%)	
	Unknown	–				189 (12.02%)	15 (19.23%)	174 (11.65%)	
Insurance status	Private	32 (20.92%)	5 (19.23%)	27 (21.26%)	1.000		–		
	Public	107 (69.93%)	19 (73.08%)	88 (69.29%)			–		
Parasite exposures	Evidence of local exposure	6 (3.92%)	6 (23.08%)	0 (0.00%)	<0.001		–		
	Travel exposure	17 (11.11%)	4 (15.38%)	13 (10.24%)	0.493		–		
	Frequent time spent outdoors		–			554 (35.24%)	29 (37.18%)	525 (35.14%)	0.111
	Foreign-born resident	77 (50.33%)	16 (61.54%)	61 (48.03%)	0.282		–		
Severe disease risk factors	Malnutrition	9 (5.88%)	0 (0.00%)	9 (7.09%)	0.359		–		
	Living with HIV	5 (3.27%)	1 (3.85%)	4 (3.15%)	1.000		–		
	Reported alcohol use	25 (16.34%)	2 (7.69%)	23 (18.11%)	0.252		–		
	Chronic asthma		–			257 (16.35%)*	13 (16.67%)*	244 (16.33%)*	0.009
Immunocompromising conditions	None	117 (76.47%)	23 (88.46%)	94 (74.02%)	0.053		–		
	Cancer	6 (3.92%)	3 (11.54%)	3 (2.36%)			–		
	Transplant recipient	1 (0.65%)	0 (0.00%)	1 (0.79%)			–		
	Inherited immune deficiency	2 (1.31%)	0 (0.00%)	2 (1.57%)			–		
	Autoimmune disease	5 (3.27%)	0 (0.00%)	5 (3.94%)			–		
	Other	21 (13.73%)	0 (0.00%)	21 (16.54%)			–		
	Cancer and autoimmune disease	1 (0.65%)	0 (0.00%)	1 (0.79%)			–		
Hospital-associated outcomes	Admitted to ICU	4 (2.63%)	1 (3.85%)	3 (2.38%)	0.532		–		
	Inpatient mortality	10 (6.54%)	3 (11.54%)	7 (5.51%)	0.375		–		

ALL-IN = A Learning Laboratory: Innovative, National, Self-Collection Study; ICU = intensive care unit; SC = South Carolina.

* Unknown chronic asthma status values included *n* = 83 (5.3%) of the entire study population, *n* = 10 (12.8%) of the positive participants, and *n* = 73 (4.9%) of the negative participants.

### Biobanked seroprevalence study.

For the seroprevalence study, a total of 1,572 serum samples were tested for *S. stercoralis* antibodies, with 55 initially testing positive and 61 yielding equivocal results. Among the equivocal cases that were retested, 23 (37.7%) tested positive. Subsequently, a total of 78 serum samples (5.0%) were classified as *S. stercoralis* antibody positive. Positive samples statistically differed by race/ethnicity (*P* <0.001), income level (*P* <0.014), and education level (*P* = 0.037) (Table 1). Specifically, positive samples were more likely to be non-Hispanic Black residents (41.0% [cases] versus 23.4% [controls]), have an income of $50,000–$100,000 (21.8% [cases] versus 13.9% [controls]), and report “unknown” levels of education (20.5% [cases] versus 11.4% [controls]). Medical histories and parasite-specific exposure knowledge were limited, although report of chronic asthma status was known and statistically lower in the *S. stercoralis* antibody-positive participants (70.5% versus 78.8%; *P* <0.009).

**Figure 2.**
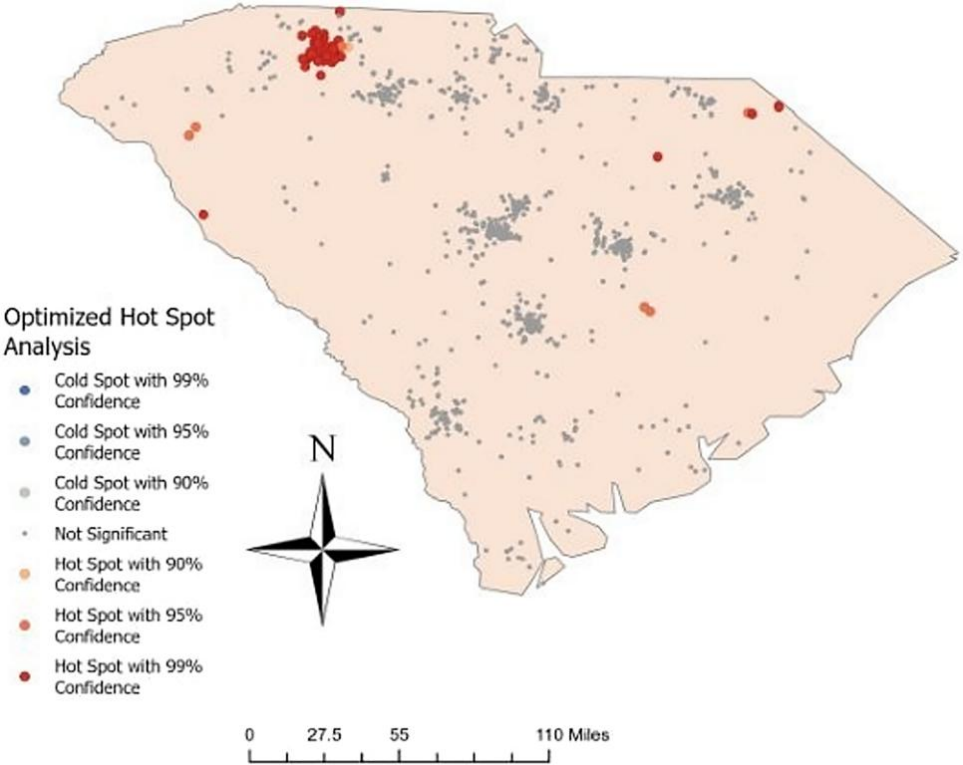
Geospatial hot spots of *Strongyloides stercoralis* human case clusters in South Carolina.

Participant samples for the cross-sectional seroprevalence study originated from all four state public health regions. Statistical hot spot analysis identified four seropositive clusters, including a prominent hot spot at the 99% confidence level in the Spartanburg, Camden, Greenwood, and Chesterfield, SC areas (Figure 2). Two additional hot spots at the 95% confidence level were noted in Anderson and central southeast SC. The rest of the state exhibited nonsignificant, dispersed patterns of both positive and negative participant serum samples.

### Follow-up of participants with a seropositive biobanked sample.

All participants from the ALL-IN SC biobank seroprevalence study who tested positive were sent invitations to take part in a follow-up confirmation study (as described in the Materials and Methods section). Fourteen of the 78 positive cases (18.0%) completed a confirmation study visit. Of the 14 prospectively tested participants for *S. stercoralis* infection, 4 tested positive (35.7%) on contemporary antibody assays; none were positive by PCR. Given the low count, univariate regressions were not performed, and trends are reported here. Demographics correlated with repeat reactive tests included being non-Hispanic White race/ethnicity (80% [repeat positives] versus 22% [repeat negatives]) and residing in an urban or suburban location (80% [repeat positives] versus 44% [repeat negatives]) (Table 2). Risk factors for repeat reactive tests included currently having cancer (40% [repeat positives] versus 0% [repeat negatives]), being less likely to consume alcohol (40% [repeat positives] versus 89% [repeat negatives]), being less likely to regularly see a medical doctor (60% [repeat positives] versus 78% [repeat negatives]), never spending time outdoors barefoot (60% [repeat positives] versus 33% [repeat negatives]), being more likely to have indoor plumbing without a septic system (80% [repeat positives] versus 44% [repeat negatives]), being more likely to work outside the home (80% [repeat positives] versus 44% [repeat negatives]), and being less likely to have a history of international travel (40% [repeat positives] versus 78% [repeat negatives]). Lastly, repeat positives were notably less likely than repeat negatives to have current parasite-related symptoms, with most repeat negatives reporting abdominal pain, diarrhea, skin rash, or breathing-related complaints.

**Table 2:** Characteristics of seropositive participants from the A Learning Laboratory: Innovative, National, Self-Collection Study (ALL-IN) at follow-up

Demographics and Characteristics	Total Unique Participant Sera Samples, *N* = 14	*Strongyloides stercoralis*- Repeat Positive Participants, *n* = 5 (35.7%)	*Strongyloides stercoralis*- Negative Participants, *n* = 9 (64.9%)
Demographics			
Age, years			
18–24	6 (42.86%)	2 (40.00%)	4 (44.44%)
25–34	2 (14.29%)	1 (20.00%)	1 (11.11%)
35–64	0 (0.00%)	0 (0.00%)	0 (0.00%)
65+	6 (42.86%)	2 (40.00%)	4 (44.44%)
Gender			
Male	6 (42.86%)	3 (60.00%)	3 (33.33%)
Female	8 (57.14%)	2 (40.00%)	6 (66.667%)
Race/ethnicity			
Non-Hispanic White	6 (42.86%)	4 (80.00%)	2 (22.22%)
Non-Hispanic Black	8 (57.14%)	1 (20.00%)	7 (77.78%)
Annual household income, $			
<30,000	2 (14.29%)	0 (0.00%)	2 (22.22%)
30,000–59,000	3 (21.43%)	2 (40.00%)	1 (11.11%)
60,000–90,000	3 (21.43%)	1 (20.00%)	2 (22.22%)
90,000–120,000	2 (14.29%)	1 (20.00%)	1 (11.11%)
>120,000	4 (28.57%)	1 (20.00%)	3 (33.33%)
Domestic property characteristics			
Urbanicity			
Urban	3 (21.43%)	2 (40.00%)	1 (11.11%)
Suburban	5 (35.71%)	2 (40.00%)	3 (33.33%)
Rural	6 (42.86%)	1 (20.00%)	5 (55.56%)
Home type			
Single unit	9 (64.29%)	3 (60.00%)	6 (66.67%)
Multifamily unit	5 (35.71%)	2 (40.00%)	3 (33.33%)
Farm property	3 (21.43%)	1 (20.00%)	2 (22.22%)
Foundation type			
Concrete slab	4 (28.57%)	1 (20.00%)	3 (33.33%)
Full basement foundation	4 (28.57%)	2 (40.00%)	2 (22.22%)
Pier and beam (concrete)	5 (35.71%)	2 (40.00%)	3 (33.33%)
Not sure	1 (7.14%)	0 (0.00%)	1 (11.11%)
Number of people in residence			
Lives alone	2 (14.29%)	1 (20.00%)	1 (11.11%)
2 people in the home	8 (57.14%)	3 (60.00%)	5 (55.56%)
3–4 people	3 (21.43%)	1 (20.00%)	2 (22.22%)
5 or more people	1 (7.14%)	0 (0.00%)	1 (11.11%)
Personal health habits			
Any chronic condition			
None	10 (71.43%)	3 (60.00%)	7 (77.78%)
Asthma	1 (7.14%)	0 (0.00%)	1 (11.11%)
Cancer	2 (14.29%)	2 (40.00%)	0 (0.00%)
Asthma and cancer	1 (7.14%)	0 (0.00%)	1 (11.11%)
Ever taken an HIV test	9 (64.29%)	3 (60.00%)	6 (66.67%)
Uses any tobacco product	1 (7.14%)	0 (0.00%)	1 (11.11%)
Drinks alcohol	10 (71.43%)	2 (40.00%)	8 (88.89%)
Uses recreational drugs	1 (7.14%)	0 (0.00%)	1 (11.11%)
Reports seeing doctor regularly	10 (71.43%)	3 (60.00%)	7 (77.78%)
*Strongyloides stercoralis* parasite-specific exposures			
Spends time barefoot outside			
Never	6 (42.86%)	3 (60.00%)	3 (33.33%)
Rarely	3 (21.43%)	0 (0.00%)	3 (33.33%)
Sometimes	4 (28.57%)	1 (20.00%)	3 (33.33%)
All of the time	1 (7.14%)	1 (20.00%)	0 (0.00%)
Home sanitation system			
Indoor plumbing without septic system	8 (57.14%)	4 (80.00%)	4 (44.44%)
Indoor plumbing with backyard septic system	5 (35.71%)	1 (20.00%)	4 (44.44%)
Outhouse without indoor plumbing	1 (7.14%)	0 (0.00%)	1 (11.11%)
Works outside the home	8 (57.14%)	4 (80.00%)	4 (44.44%)
Regularly spends time outdoors	13 (92.86%)	5 (100.00%)	8 (88.89%)
History of international travel	9 (64.29%)	2 (40.00%)	7 (77.78%)
*Strongyloides stercoralis* parasite-specific symptoms			
Abdominal pain	3 (21.43%)	0 (0.00%)	3 (33.33%)
Diarrhea or constipation	5 (35.71%)	0 (0.00%)	5 (55.56%)
Skin rash	5 (35.71%)	1 (20.00%)	4 (44.44%)
Dry cough, throat irritation, shortness of breath, and/or wheezing	2 (14.29%)	0 (0.00%)	2 (22.22%)

## DISCUSSION

This is one of only a handful of studies in the last 50 years to demonstrate evidence of active, autochthonous *S. stercoralis* infection foci in the southeastern United States.^9^^,^^10^^,^^18^ Using a two-pronged study design encompassing retrospective analysis of hospital records and biobanked sera and a prospective follow-up study, these results provide irrefutable evidence that an important neglected parasitic infection is causing clinical disease in twenty-first-century SC. This study identified that ∼5% of state residents have *S. stercoralis* antibodies, indicative of recent exposure or current infection. A prospective follow-up from this biobanked serosurvey found that 18% were repeat reactive up to 2 years later, demonstrating undiagnosed and untreated active infections. Further, medical chart abstractions suggest that up to 17% of tested patients are positive for infection, highlighting that this infection is not rare among those with clinical risk factors. Lastly, this study found that 29% of positive *S. stercoralis* persons had evidence of locally acquired infection (6 from the retrospective chart review [6 of 26] and 3 from the prospective follow-up study [3 of 5]). Although overall positive *S. stercoralis* numbers were low, the impact of this study is notable, with active, ongoing infection leading to clinical disease occurring in the modern-day United States.

Among the most striking of the study’s findings were the sheer numbers of positive cases who reported no international travel and had evidence of autochthonous infection. In the retrospective chart abstraction, 85% (*n* = 22 of 26) of positive patients reported no recent travel history, and several patients’ charts had a provider-determined “local exposure” (i.e., gardening, outdoor hobbies, or having pets as reported by clinicians). Unfortunately, the biobanked serosurvey’s associated health survey did not include questions about international travel; however, the prospective follow-up survey did. Notably, none of the 14 prospectively followed participants with a prior positive serology report reported foreign birth, and 3 of the 5 repeat positive participants reported no international travel in the last decade. Additional occupational and parasite exposures were noted among both groups, particularly that in both cohorts, confirmed cases were more likely to work outside. Lastly, the potential for local transmission is further substantiated by a recent study from external colleagues (not associated with the current study) who reported STHs in SC-collected soil.^8^ Collectively, these data suggest that a small number of residents are likely acquiring this neglected parasitic infection from their immediate environment.

Although not a representative sample of SC’s statewide population, the banked serosurvey and subsequent geospatial analyses suggest an *S. stercoralis* hot spot in the upstate region, which is ecologically dominated by the Appalachian Mountain range. This is consistent with the known historical and proposed modern-day range of *S. stercoralis* in the United States, which includes other locations along this cordillera.^2^ Cross-sectional studies from Kentucky, Alabama, and Mississippi published in the 10 years prior to this article support the likelihood that “Appalachia” cultural practices leading to parasitic presence continue today, despite the cessation of the historic Rockefeller Sanitary Commission studies.^7^^,^^9^^,^^10^^,^^19^ Thus, although far from conclusive, it is possible that this study’s findings are not restricted to SC alone.

If *S. stercoralis* does have ongoing local transmission but prevalence is low, this begs the question of how the parasite is able to complete its life cycle with so few human infections. Potential answers are 4-fold. First, it is possible that this study’s findings are an underestimation of SC’s true *S. stercoralis* burden given that the highest-risk populations (rural, poor sanitation infrastructure, etc.) are often the most difficult to engage, especially given the historical medical atrocities conducted on southern populations of color and contemporary distrust of the medical and scientific communities.^20^ Second, *Strongyloides* spp. can be a long-lived human parasite, known to persist in the human gut for decades given its ability to autoinfect. Fewer human infections are, therefore, required to maintain its ongoing presence in a population. Third, *S. stercoralis* is unique among human parasitic nematodes in that it can complete its life cycle entirely independent of a human host—meaning that it can persist in the environment long term, even if few human hosts are found. Lastly, there is evidence that some *S. stercoralis* found in domesticated dogs is genetically identical to that in human hosts, suggesting that zoonotic transmission is possible, with dogs as a nonhuman reservoir.^21^ All of these factors support the possibility of local *S. stercoralis* endemicity, even in low-burden settings.

Local providers’ general unfamiliarity with *S. stercoralis* is an ongoing barrier to greater detection and control of this parasite in the state. During the chart review analysis, *S. stercoralis* was sometimes confused with pinworm (*Enterobius vermicularis*), and the incorrect antiparasitic medication was prescribed. Although this confusion is comprehensible given the rarity of the parasite and providers’ limited experience with helminthic infections in general, a missed *S. stercoralis* case can result in severe patient consequences. Persons living with HIV, cancer, or other immunosuppressive conditions are particularly at risk for hyperinfection syndrome. Those at greatest risk are persons living with chronic infection that are newly initiating immunosuppressant medications; therefore, it is imperative that family medicine doctors, internists and oncologists consider STH panel testing for at-risk populations living in the southern states with historic transmission.^10^ Further, missed cases contribute to ongoing transmission; thus, identification of veritable cases not only benefits the immediate patient but also, their family (in the setting of expanded family testing) and those in the broader local community. Greater awareness and ongoing surveillance strategies are necessary to better detect and control *S. stercoralis* transmission in SC and nearby states.

There are a few study limitations worth discussion. First, the biobanked serosurvey used a convenience sampling design, which was not representative of SC’s population. The original study focused outreach on three marginalized communities (rural populations, lower socioeconomic populations, and populations of color), which are independent at-risk groups for infection that may have biased the seroprevalence results. Robust, representative samples to validate state seroprevalence estimates are warranted. Second, the follow-up prospective sampling and survey results may be biased because of low participation rates and overall low counts. These results should be interpreted with caution. Third, detailed exposure and travel histories were not available given the study designs used, which limits the ability to confirm infection origin. Lastly, none of the participants from the three studies had positive PCR results, which placed diagnostic reliance on serologies. Serologies are not perfect indicators of active infection, nor do they provide *Strongyloides* species confirmation. To compensate for this potential, the authors performed multiple rounds of serologic testing and used a conservative positive diagnostic test definition (2+ plate wells with a positive test result).

## CONCLUSION

In 2017, Sanders and Goraleski^22^ wrote an editorial in this same journal highlighting “sober reminders that tropical medicine and hygiene has relevance even within the border of the United States.” This article serves as a contemporary reminder to public health and medical providers who serve the southern United States’s vulnerable populations that neglected infections are still occurring, and we have far to go to mitigate and eliminate these forgotten diseases. This multiapproach study provides concrete evidence that autochthonous *S. stercoralis* infections are occurring in SC and have medical impact. Future studies are warranted to eliminate this chronic parasite that can exacerbate cognitive and physical disabilities and hinder one’s upward economic mobility.
